# Job vacancy at the European Centre for Disease Prevention and Control (ECDC)

**DOI:** 10.2807/1560-7917.ES.2023.28.38.2309215

**Published:** 2023-09-21

**Authors:** Eurosurveillance editorial team

**Affiliations:** 1 European Centre for Disease Prevention and Control (ECDC), Stockholm, Sweden

The European Centre for Disease Prevention and Control (ECDC) invites applications for the following position:

 **Scientific Officer Bioinformatics**

The application deadline expires on 17 Oct 2023.

 For more information, please visit the ECDC website: https://erecruitment.ecdc.europa.eu/?page=advertisement

